# Reproducibility in Human-Robot Interaction: Furthering the Science of HRI

**DOI:** 10.1007/s43154-022-00094-5

**Published:** 2022-10-22

**Authors:** Hatice Gunes, Frank Broz, Chris S. Crawford, Astrid Rosenthal-von der Pütten, Megan Strait, Laurel Riek

**Affiliations:** 1 Department of Computer Science and Technology, University of Cambridge, Cambridge, UK; 2 Interactive Intelligence Group, Delft University of Technology, Delft, The Netherlands; 3 Department of Computer Science, University of Alabama, Tuscaloosa, AL USA; 4 Department of Society, Technology, and Human Factors, RWTH Aachen University, Aachen, Germany; 5 Department of Computer Science, University of Texas Rio Grande Valley, Brownsville, TX USA; 6 Department of Computer Science and Engineering, University of California San Diego, San Diego, CA USA

**Keywords:** Reproducibility, Human-robot interaction

## Abstract

**Purpose of Review:**

To discuss the current state of reproducibility of research in human-robot interaction (HRI), challenges specific to the field, and recommendations for how the community can support reproducibility.

**Recent Findings:**

As in related fields such as artificial intelligence, robotics, and psychology, improving research reproducibility is key to the maturation of the body of scientific knowledge in the field of HRI. The ACM/IEEE International Conference on Human-Robot Interaction introduced a theme on Reproducibility of HRI to their technical program in 2020 to solicit papers presenting reproductions of prior research or artifacts supporting research reproducibility.

**Summary:**

This review provides an introduction to the topic of research reproducibility for HRI and describes the state of the art in relation to the HRI 2020 Reproducibility theme. As a highly interdisciplinary field that involves work with technological artifacts, there are unique challenges to reproducibility in HRI. Biases in research evaluation and practice contribute to challenges in supporting reproducibility, and the training of researchers could be changed to encourage research reproduction. The authors propose a number of solutions for addressing these challenges that can serve as guidelines for the HRI community and related fields.

## Introduction

By reproducing, comparing, and evaluating the effectiveness of solutions to technical or scientific problems, work on reproducibility and benchmarking has played a significant role for pushing the state of the art in several fields relevant to human-robot interaction (HRI), including computer vision, machine learning, and artificial intelligence. This topic is especially important to HRI, as it is also a field of human-focused research that is at risk of a similar reproducibility crisis as has been identified in fields such as psychology.

Despite the increasing encouragement and pressure for reproducibility in other fields, the robotics field in general and the HRI field in particular have fallen behind in following this trend [1]. Lacking theoretical principles and practical resources to reproduce results has significant implications for the science of HRI: (1) it is not possible to build on existing body of research and extend the state of the art, (2) it is not possible to objectively evaluate the state of the art for the various themes or sub-fields of HRI, and (3) these in turn negatively impact the societal take-up and industrial exploitation of HRI research outcomes. Some of these issues are discussed in more detail in [2] for the robotics field at large.

In addition to the abovementioned challenges, issues related to generalizability (across contexts, populations, platforms, etc.) make reproducibility even more relevant for furthering the science of HRI. We know, for instance that, findings of existing literature may not necessarily generalize to other populations [3]; perceptions of robot designs and uses can vary significantly between stakeholders (e.g., across cultural affiliations [4], between children and young adults, [5, 6], and between young adults and older adults [7]); and affinity for/aversion to a robot can vary significantly across designs (e.g., uncanny valley phenomenon [8]).

**Fig. 1:**
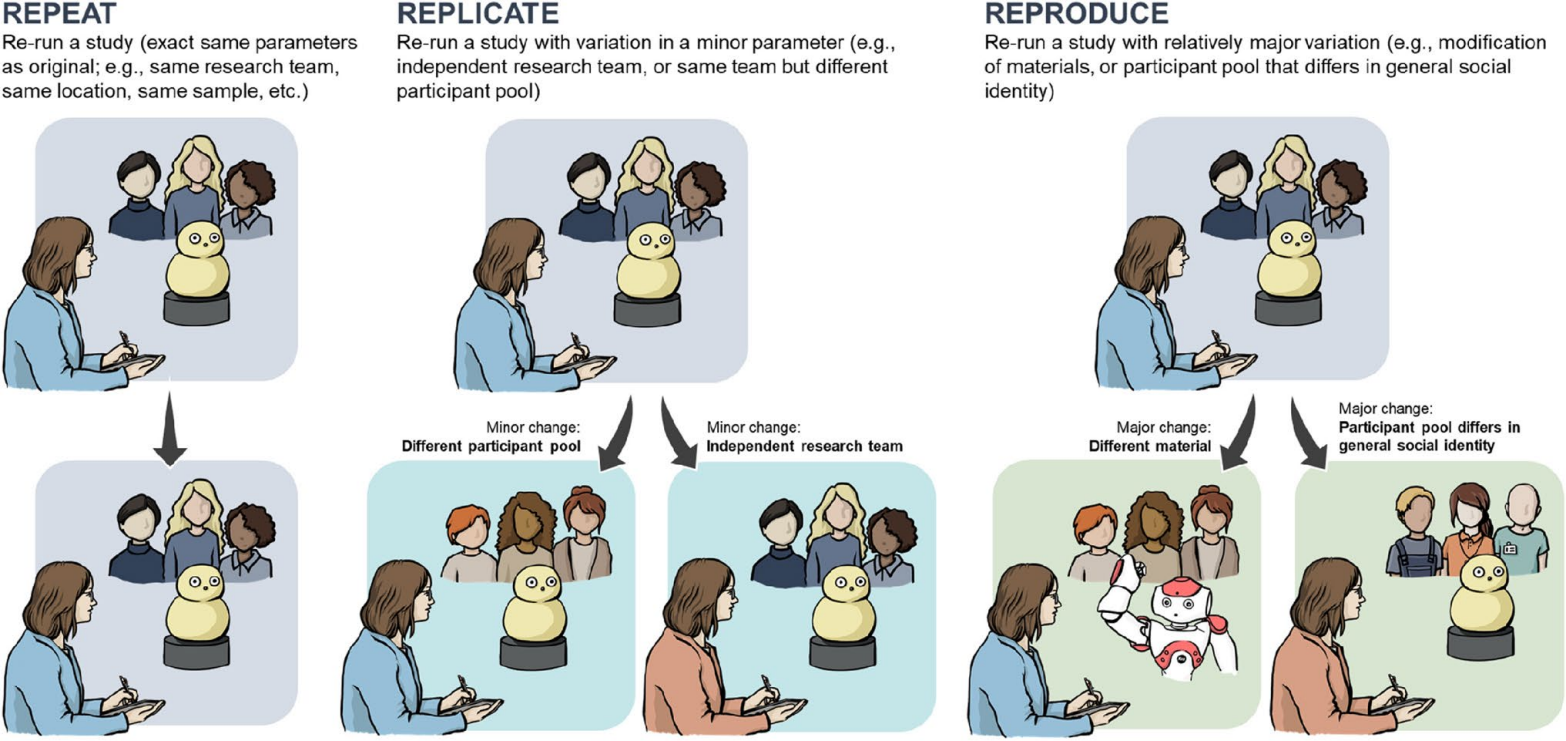
Illustration of the differences between *repeat*, *replicate*, and *reproduce* in the context of reproducibility in HRI

These motivations were the major drive for two of this paper’s authors, Riek and Gunes, to create a new theme entitled “Reproducibility of Human-Robot Interaction” for the 2020 ACM/IEEE International Conference on Human-Robot Interaction (HRI 2020) in their roles as Program Chairs. They invited the other authors to serve as Section Chair (Strait) and Area Chairs (Rosenthal-von der Pütten, Crawford, Broz). In these unprecedented times of COVID-19 and lockdowns, when it is extremely challenging to undertake face-to-face human-robot interaction studies at universities and research institutes, the necessity of reproducibility and the importance of artifacts for reproducibility have become more relevant than ever. As the science of reproducibility in HRI continues to evolve, it is helpful to define key terminology which will be used throughout this article. Table 1 presents terms and their common purposes are illustrated by Fig. 1.

This paper is organized as follows. In the “Reproducibility in HRI” section, we present what reproducibility in the context of HRI entails, and how this was introduced and brought to life as a theme at HRI 2020. The “Technical Challenges to Supporting HRI Reproducibility” section focuses on the technical challenges to supporting HRI reproducibility. The “Bias in HRI Research Evaluation and Practice” section introduces the challenges related to bias in HRI research evaluation and practice. The “Educating and Training Reproducibility Researchers” section discusses how to educate and train reproducibility researchers, while the “Summary and Conclusion” section provides suggestions for supporting reproducibility in HRI. The “Summary and Conclusion” section concludes the paper.

**Table 1:** Key terminology and common purposes for reproducibility in HRI

Repeat	Re-run a study (exact same parameters as original; e.g., same research team, same location, same sample size) for the purpose of validating or characterizing the reliability of measurements.
Replicate	Re-run a study with variation in a minor parameter (e.g., independent research team or same team but different participant pool).
Reproduce	Re-run a study with relatively major variation (e.g., modification of materials or participant pool that differs in general social identity). May be further specified as direct or conceptual (as defined by [9]).
Direct reproduction	Aim to obtain the same results from an independently conducted study using procedures and methods as closely matched to the original study as possible in order to evaluate the reliability of a previously observed finding.
Conceptual reproduction	Aim to obtain the same results from an independently conducted study where procedures and methods are systematically varied in order to build upon prior evidence to understand under what conditions and for who a finding holds true.
Artifact	Aim to introduce a novel contribution as an enabler to reproducibility, replicability, and/or re-creation of research. Could be software, hardware, data sets, protocols, evaluation measures, etc.

## Reproducibility in HRI

Compared to other fields, where reproducing code/software or providing new datasets (created and obtained with Web crawling) is the main goal, reproducibility in HRI needs to take into account additional considerations along several themes, which align to the conference organization itself: (1) User Studies (data and analysis in lab and in-the-wild settings); (2) Technical Advances (algorithms, interface technologies, and computational methods); (3) Design (of new robot morphologies and appearances, behavior paradigms, interaction techniques and scenarios, and interfaces supporting interaction experiences or abilities for robots); and (4) Theory and Methods (contributing to the understanding and study of fundamental HRI principles underlying interaction paradigms, theoretical concepts, or new evaluation methodologies).

In light of these, HRI 2020 described the Reproducibility theme as targeting research that makes a contribution supporting the science of HRI via reproducing, replicating or re-creating prior HRI/HRI-relevant work, and artifacts for HRI research, to help the community build a strong and reliable evidence base. As the theme was new, we provided additional descriptions and details in the call for papers (CFP)^1^ on how to reproduce HRI work depending on the type of work undertaken (quantitative vs. qualitative). For reproducing prior quantitative HRI work, reproductions could span quantitative work across the spectrum of HRI themes — Studies, Technical, Methods, or Design (e.g., the original findings were obtained through primarily quantitative methodologies). Here are a few examples that were provided of conceptual replications per theme area:

- **Studies/Design Conceptual Reproduction Example**: If an author’s goal is to see whether behavior previously observed with robot *R* similarly manifests with other robots, they might vary the platforms but employ the same method. If they are also curious about how the methods used in the original study affected the results, they may vary the methods used in the original study;
- **Technical Conceptual Reproduction Example**: If an author’s goal is to see if a teaming algorithm presented in a prior paper yields the same results on experiments conducted on other robot platforms, they would vary the robot platform but employ the same method;
- **Theory and Methods Conceptual Reproduction Example**: If an author’s goal is to see whether a theory or method presented in prior work as being suitable for culture *C* also holds true in cultures $C^1$ and $C^2$, they would vary the cultural context but employ the same method and/or robot.

In the CFP, we suggested that when authors are reproducing, replicating, or repeating quantitative work, they should follow the guidelines developed by the US National Science Foundation and Dept. of Education on how to design, conduct, and report such studies [9]. We also encouraged authors to report negative results of reproduction, which is a key aspect of furthering the science of HRI (e.g., the undertaken work fails to reproduce or replicate another study’s findings).

Reproducibility within the context of **qualitative or design-focused** HRI work seeks to explore an HRI paradigm within a new culture or context, or re-create or implement designs created by another. This work could be framed as case studies, field reports, or updated design guidelines, and authors were encouraged to clearly describe lessons learned and best practices.

For artifact submissions, authors were encouraged to include a detailed description of the artifact introduced, proposed, or implemented, as well as information about how it was novel and different from other existing artifacts, and a link to an anonymized, live version of the artifact at time of submission for review.

### HRI 2020 Reproducibility Theme

The first Reproducibility theme at HRI 2020 showed promising variety in how the community is beginning to address these topics. The selected papers describe studies ranging from conceptual reproduction to replication. These papers also include qualitative analysis and design work to support reproducibility, software re-use, the use of online studies in research reproduction, and a range of artifacts supporting reproduction (that were submitted as part of their scholarly contribution).

Strait et al. [10] describe a conceptual reproduction of a study on the Joint Simon effect (JSE), replicated at three geographic locations with differing cultures. The paper also describes the use of toolkits to design the experiment software system to be deployed identically at multiple sites. The authors found evidence that the JSE generalizes across population and setting but did not find an amplifying effect of perceived robot agency.

Sandygulova et al. [11] replicate a prior study that is a conceptual reproduction of a robot-supported Learning by Teaching paradigm, implemented by extending an existing software system to support learning to write Kazakh. The study demonstrated the success of the paradigm for this learning task, and a gender effect was found in which style of robot assistance was most effective.

Kubota et al. [12] presented JESSIE, a robot behavioral specification system, which allows people with no prior programming experience to write complex, dynamic control software for robots using only paper cards and a camera. The system was demonstrated on two different physical robot embodiments and all supplemental materials are released as open source to support reproducibility. JESSIE enabled clinicians with no prior programming experience to author cognitive therapy sessions for delivery by a robot.

Pereira et al. [5] replicate a prior study evaluating a joint attention system using child participants rather than adults. Additionally, they reproduce this prior work by adapting it into an online study to test whether effects on perceived social presence extend to observers of the system. They found evidence that the joint attention system led to greater perceived social presence by adult observers but did not find that this result generalized to the child-robot interaction context.

Li et al. [13] conduct a direct reproduction of a “ghost-driver” experimental paradigm in Europe, comparing results to those in other parts of the world. They also produce and evaluate a conceptual reproduction of this paradigm as an online study. They found that the hidden driver paradigm is valid in Europe and confirm prior results relating responses to self-driving cars to group size. But they also found the level of belief in a car’s autonomy to be lower online than in person.

It was also positive to see papers submitted to other themes which included support for research reproduction. Examples include video studies whose authors included their experimental stimuli as supplemental materials to their publications [14, 15]. Additionally, at least one paper’s authors publicly released their full dataset and a software specification of the neural network architecture used in their publication [16].

While the HRI 2020 submissions were encouraging, the track revealed some areas for improvement and a need to increase community engagement with issues of reproducibility. These included (1) terminology confusion; (2) experimental design and evaluative challenges; (3) a lack of submissions which reproduced design/qualitative work; (4) a lack of repeatability studies; and (5) few systems submissions.

## Technical Challenges to Supporting HRI Reproducibility

Many HRI researchers leverage robotics systems that involve both hard and soft components. Hard components are often constructed of rigid materials such as metal, wood, and plastic. Furthermore, solid component designs can range from off-the-shelf (i.e., commercial robots) to highly custom parts. On the other hand, soft components are usually constructed with data and code. HRI researchers are often tasked with configuring and integrating these hard and soft components, which can lead to technical challenges. Key common challenges related to system-related challenges and software artifacts are discussed below in the “Systems-Related Challenges” and “Software Artifacts” sections.

### Systems-Related Challenges

HRI systems are often acquired through various approaches. One method involves obtaining the hardware components pre-assembled via a commercial vendor. With this approach, the researcher is not required to construct or configure individual rigid pieces. An additional option involves acquiring a kit with several parts and instructions explaining the steps to configure a robot. HRI researchers seeking to build highly novel robotics systems may require custom solutions that are not covered by commercially available hardware. These approaches present unique challenges that are discussed further in the following subsections.

#### Commercial Robots

Preassembled commercial robot platforms are commonly mass-produced. As a result, these robots are ideal for direct reproduction studies that require identical physical robot designs. However, leveraging commercially available robots may come with the trade-off of reduced flexibility due to the frequent use of hard rigid plastic [17]. Consequently, commercial robotic hardware solutions may present challenges if a team wishes to change the robot’s physical design systematically. Furthermore, the cost associated with commercially available robots could limit access to existing platforms.

#### Robot Kits

Unassembled robotic hardware requires users to connect multiple physical components before using the robot. This approach is common when one seeks to build a predesigned or custom robot. Robotic kits are often used to facilitate the process of shipping and assembling predesigned robots. Similar to the commercial robot platforms mentioned previously, these kits are usually mass-produced which could benefit direct reproduction research. The modular nature of robotic kits may allow for easier customization in comparison to preassembled robotic platforms alternatives. As a result, the kit approach may provide a positive example of ways to support conceptual reproduction research. However, clear and user-friendly assembly instructions may be required to ensure that all assembled robots are similar to support direct reproduction studies.

#### Custom-Designed Robots

Preassembled commercial robot platforms and robotic kits may be less effective when researchers seek to explore an entirely custom robot design. While research featuring new custom robot designs plays a crucial role in expanding our knowledge, it could be challenging for a new team to conduct direct reproductions of studies featuring complex custom robot designs. Unlike the robotic kit approach, this work is usually not accompanied by step-by-step assembly instructions.

### Software Artifacts

Software plays a critical role in assisting researchers in conducting HRI science. Several software components exist that provide access to underlying operating system and communication primitives. As a result, HRI developers may choose from various development approaches. These development strategies can range from creating custom software with robot specific software development kits (i.e., NAOqi C++ SDK – http://doc.aldebaran.com/2-5/dev/cpp/)^2^ to using platform-specific development applications (Choregraphe – http://doc.aldebaran.com/1-14/software/choregraphe/choregraphe_overview.html)^3^. Additionally, developers may leverage robotic middleware to create highly complex robotic applications.

Practices around the use and (lack of) distribution of research software remain a barrier to reproducibility of research involving robots. Only 16 of the 800 papers published in the 2017 Proceedings of the IEEE International Conference on Robotics and Automation referenced source code that was successfully built and run; just one of the 16 papers referenced source code that could be run off-the-shelf (the other 15 required extensive, non-automateable work to comprehend, supplement, and execute their code) [18•].

Source code and data designed for robotics research are packaged in different formats. For example, vendors often provide commercial robotics software for specific hardware platforms. This software typically supports a limited set of pre-defined functions that facilitate communication between a device and the robot. Software development kits (SDKs), on the other hand, usually allow researchers to extend a robot’s capabilities beyond pre-defined robot behaviors. When addressing more complex objectives, HRI researchers often leverage middleware systems that generally offer higher levels of customization. The challenges and trade-offs involved with each of these approaches are discussed in the following subsections.

#### Commercial Solutions

Commercial robotics software solutions often enable end-users to create robotics applications without requiring specific knowledge of low-level components (i.e., device drivers). A common approach is to provide graphical user interface (GUI)–based development environments that assist developers with managing robotics components (e.g., sensors and actuators). The design of these commercial-based solutions lead to robotics applications that may be easily shared across users using the same version of the development environment and robot. As a result, this approach may offer promising support for direct reproductions. However, there are several trade-offs that may lead to various limitations. In particular, the cost associated with some commercial robotics software solutions may present barriers. Furthermore, commercial solutions often leverage proprietary software which could make it difficult to implement novel custom behaviors for conceptual reproductions.

#### SDKs

Robotics SDKs provide tools, documentation, and relevant examples that aid developers in creating robotic applications. These kits often support functions related to communication and control via libraries written in multiple programming languages. Contrary to the GUI-based approach, SDKs may provide better support for application customization. SDKs can be especially useful in designing conceptual reproductions. This is particularly true when the application’s source code is maintained and shared via a source code management platform. Nevertheless, reproduction efforts may be hindered if relevant source code is not shared or preserved. It is common for simple applications to only require one SDK. However, complex applications may include multiple hardware components and SDKs. Developers often use middleware in place of individual SDKs to manage these more complex applications.

#### Middleware

Robotics middleware provides frameworks that assist developers in managing complex robotic systems. These platforms commonly abstract low-level functions and provide tools to help with the integration of new hardware components. Popular robotics middleware such as ROS [19] and Player [20] has been leveraged by several researchers to design robotics applications. This collaborative approach has led to large ecosystems that can be beneficial for reproduction and replication efforts. However, missing configuration details such as required datasets could impede reproducibility efforts. Lier et al. [21] recently developed the Cognitive Interaction Toolkit (CITK) to address these issues. Recently, researchers have also developed frameworks in response to common challenges faced when designing multimodal interactive systems [22–24]. Continued work on these types of frameworks may facilitate HRI replication and reproduction due to their focus on functions related to voice and gestures. However, additional work is needed to understand the most effective ways to leverage frameworks to promote and support reproducibility in HRI.

## Bias in HRI Research Evaluation and Practice

Another challenge faced in supporting reproducibility for HRI is bias. These biases are discussed across many fields which intersect with HRI (e.g., Computer Science and Engineering, the Social Sciences, Arts and Humanities), see [25•] for recent reviews. Here, we focus on two types of bias: (1) HRI research evaluation bias and (2) social systemic bias and its affect on HRI research practice.

### HRI Research Evaluation Biases

HRI researchers undergo a range of evaluations of their work including paper reviews, grant reviews, and job applications. Across each of these, multiple research biases can be introduced by evaluators. Part of the challenge is the multidisciplinarity of the field – it includes the technical and non-technical, the quantitative and the qualitative, the mechanistic and artistic. Evaluators bring the evaluative lenses of their respective fields to judge HRI work, which can, at times, be problematic.

After over 15 years of the HRI conference, where many of this article’s authors have served on the Program/Organizing committee (PC/OC) in various roles, combined with editorial service on relevant journals (e.g., THRI, SORO), we have thus amalgamated a list of evaluative biases our field continues to face.

#### Null Hypothesis Testing (NHT)/Normative Statistics Bias

HRI evaluators who come from the psychological and cognitive sciences tend to expect HRI research to employ NHT research methods (e.g., quantitative user studies). While for some HRI research this perspective makes sense, it is often problematic with regard to other types of contributions. For example, much exploratory design work and theoretical algorithmic work in HRI does not yet have a hypothesis to test, yet may still provide a valuable contribution to the field. Furthermore, many quantitative fields, ranging from statistics to epidemiology, have encouraged alternate methodological designs, and suggest moving away from normative statistics due to p-hacking and other serious methodological concerns [26, 27].

However, many HRI researchers feel pressure to “add a [NHT] user study,” which can often result in an attempt to appease NHT-lensed evaluators that fall flat. Neither technically nor qualitatively trained researchers tend to have an NHT perspective, so may struggle with fitting their work into that box. Furthermore, few are trained in human experimental design and normative statistical analyses.

At HRI 2015, the PC chairs first introduced the idea of review tracks, which included separating “User Studies” into qualitative and quantitative. They also separated out the Design and Technical tracks. While these changes have helped ease bias, 5 years later, we find HRI authors still face pressure to apply NHT approaches across all tracks, which can have deleterious effects on the review process.

Thus, NHT-bias is perhaps one of the more challenging biases HRI reproducibility faces for several reasons. First, authors may be attempting to reproduce/replicate problematic work, which only serves to propagate the biased findings. Second, the very concept of reproducibility is often framed as reproductions of NHT/quantitative work, which excludes re-creations (of HRI design work), or re-implementation/re-use of artifacts, etc.

#### Sample Size Bias

Another bias many HRI evaluators have is that work must include at least *n* participants, where *n* is some particular number, else the research is considered unworthwhile. This too is very much NHT-research centric, and can further exacerbate the aforementioned issues with regard to reproducibility and field-inclusivity.

#### Novelty Bias

HRI evaluators from all perspectives tend to expect and favor HRI submissions which present completely novel ideas. This means two key types of HRI Science, namely, reproducibility and systems-building, suffer despite being essential to the Science of HRI. At HRI 2020, the PC Chairs introduced the Reproducibility track particularly to help address this issue, though systems papers, unfortunately, still suffered.

#### Positive Results Bias

Just like evaluators in many other disciplines, HRI evaluators tend to favor papers that report positive results, i.e., papers with results supporting a hypothesis, as well as papers reporting novel studies. Thus, replication studies that fail to reproduce prior significant results are especially at a disadvantage regarding their evaluation as has been shown in experimental studies in real conference peer-review processes [28]. Regardless of the study being original or a replication, every research outcome that is based on rigorous scientific work is worthy of publication and indeed should be published. Not doing so is non-appreciative of the time and resources invested and in turn may lead to wasting more resources invested in a research idea that has been proven not to show the assumed effect. It would be difficult to state that one understands the field of research when a great part of research conducted in this field is ignored, because the non-positive results set out the limitations and boundaries of that research field.

#### Physical Robot Bias

Many HRI evaluators tend to favor research which involves “real robots,” e.g., physically embodied robots. HRI research involving simulations, video-based studies, picture-based studies, and thought experiments tends to be devalued in the evaluative process. While this perspective presents several social systemic biases (see 4.2), it also makes little sense given the COVID-19 pandemic, where physical robot studies have become nearly impossible for most researchers to conduct [29]. It is particularly important to consider this bias with regard to HRI reproducibility – it can be difficult for researchers to obtain non-standard robots, so they may be forced to conduct their reproductions using alternate means.

### Societal Systemic Biases and Their Affect on Research Practice

A range of social systemic biases also affect the practice of HRI science, and can also introduce biases when considering HRI Reproducibility work. These are outlined briefly below.

#### Classism Bias

Underlying the aforementioned physical robot bias is a classist bias that research which does not conform to the standard of an in-person study with a physical robot is subpar. Unfortunately this perspective serves a field gatekeeper, where only well-funded HRI researchers, with access to physical robots, programmers, and a participant pool whom can be well-compensated, can produce HRI research at an acceptable level of quality. This perspective prevents new researchers from entering the field, particularly those from less-resourced institutions. Given a new researcher’s foray into HRI may likely be a form of reproducibility, this bias can be problematic.

#### Racial, Ethnic, Cultural Sampling Biases

Many published studies in HRI suffer from sampling bias, reporting convenience sampling from “WEIRD” (Western, Educated, Industrialized, Rich, and Democratic) populations [30]. This is problematic because it is difficult to infer if conclusions drawn from many HRI studies hold for other populations. While a challenge for the field overall, it presents an excellent angle of opportunity for HRI Reproducibility to address.

#### Robot Morphology Biases

Much prior work demonstrates that humanoid robots are largely racialized as white or Asian, and are often hyper-feminized or hyper-masculanized [31, 32]. This is problematic because it reinforces systemic societal biases, which impacts study results. Thus, when conducting reproducibility studies, it is important for HRI researchers to be cognizant of this, and consider replacing racialized/feminized robots in their study designs.

## Educating and Training Reproducibility Researchers

When we want to promote reproducibility work, we have to reconsider how we set out our research endeavors and how we train young researchers. We need to embrace “failure,” acknowledge that reproducibility is important to and can be performed in every discipline, and change how we value, evaluate, and review research works.

### Embrace “Failure”

Researchers fear the alleged “failure” of their experiment, meaning they might not find their hypothesis supported with a (statistically significant) effect. Indeed, this is not a failure; it is a noteworthy study result that should be presented as such so that the community can learn from it (e.g., this manipulation has no effect).

Why is it that students conducting an experiment or interview for a Bachelor, Master, or Doctoral thesis are utterly disappointed when their $p-value$ is above .05 or their main hypothesis is not supported? They experience it as failure, (i) because their instructors frame it as such, (ii) because university lectures mostly present studies with statistically significant results, and (iii) because they read mostly papers with statistically significant results when preparing their theses. We need to train ourselves to be brave and proudly present all of our scientific work no matter whether it has produced a statistically significant effect or supported our hypothesis. Regardless of the discipline, the method of research, and the result: any research endeavor that has been conducted with scientific rigor should be reported and consequently it should be valued by the research community.

### Acknowledgement of the Importance of Reproducibility

Reproducibility is important to and can be done in every research discipline. Although across disciplines reproduction can take on different forms, it is always possible. Not only can an experimental study be reproduced, but we could invite another group to perform a second conversation analysis on the same data set, we can ask another philosopher to solve the same ethical problem using the tools, or present the same requirements for a new technology design to another design scholar. However, the education and training needed can vary across disciplines.

#### Quantitative Studies

Researchers in quantitative research fields, especially psychology, are already aware of the need for reproduction. Still, reproducibility is rather a side note in educational programs. It should be made a central part of methods classes and could be fostered, for instance, by encouraging students to replicate studies for their Bachelor’s or Master’s thesis. Moreover, researchers have to invest into meta-analyses, combine the results of multiple studies addressing the same research question, and use statistical approaches to derive a better estimate of the real (yet unknown) effect. Meta-analyses are, however, only conceptually covered in advanced statistical courses in specific study programs (e.g., psychology). Hence, more training programs on how to actually set out and conduct a meta-analysis are needed.

#### Qualitative Studies

Qualitative research can also be reproduced or replicated. But some qualitative studies or research projects may follow interpretive research methods where the study conditions are impossible to recreate (such as ethnographic research projects or participatory observations where the researcher him/herself is part of the research project). But what can be done is increase transparency, allowing others to evaluate the validity and reliability of research outputs and potentially reproduce the findings. Qualitative researchers need to describe the method(s) used, the research setting, and the sampling procedures, and should document interactions with participants (for a guide, see [33]). Qualitative studies should aim for systematizing their field’s research results, for instance, with narrative reviews.

### Pre-registration

In order to address publication bias towards significant findings, journals have increasingly set out to offer the submission type of registered reports or offer incentives for submitted pre-registration studies with so-called open science badges. While pre-registrations are usually performed via open platforms such as OSF (Open Science Framework) that create a time-stamped non-modifiable public record of the study and analysis plan before the data is collected and lack a review process, registered reports submitted to a journal are subject to peer review.

Some of the advantages of pre-registration are that it shifts attention from the results to the research questions and the methods used to address this question, thereby reducing publication bias. Pre-registration works against scientific misconduct such as adding or deleting observations in order to achieve some significant result or fishing for results by creative use of statistical tests. By pre-registering their studies, researchers can signal that the research was not driven by the desire to produce a significant result (c.f. https://blog.oup.com/2014/09/pro-con-research-preregistration/).

However, pre-registration potentially comes at a cost. How close must a researcher keep to the pre-registered report and will deviations be “punished” with rejection? What if a better measurement of the main study construct has become available, or the intended participant pool turns out to be unsuitable for the study? Wouldn’t it be better to revise the study design? When researchers fear punishment, they might perform sub-optimal research by choosing to faithfully stick to their registry for the sake of paper acceptance. Especially in the young field of HRI, predefined measures may be supplemented by analysis of other behaviors due to interesting observations made while conducting the study. Pre-registration should not hinder us to perform exploratory analyses that help gaining new insights or developing theories for HRI. An optimal strategy would be to foster pre-registrations without restricting the researchers’ creativity (e.g., additional exploratory analyses) or expertise regarding the optimal design (e.g., changes that actually improve the study).

Pre-registration is on the move, and despite appearing to be rather developed in psychology and the life sciences but scarce in the fields of engineering and computer science, there are recent efforts to introduce this notion to the machine learning field as an alternative publication model for research^4^. These efforts are encouraging; however, the reality remains that according to the Open Science Center: “Currently, 260 journals use the Registered Reports publishing format either as a regular submission option or as part of a single special issue. Other journals offer some features of the format.”^5^. Relevant to the field of robotics, there are only two of the 260 journals, namely *Human Behavior and Emerging Technologies* and *Human-Media Interaction* sections of Frontiers in Computer Science and Frontiers in Psychology. HRI conferences and journals might consider including pre-registration options into the portfolio of submission types.

### Rethink and Restructure Guides for Authors, Reviewers, and Editors

In an attempt to increase transparency and facilitate reproducibility of the published work, a good share of journals and conferences already encourage or even request authors to go “open” and share methods and data-sets as well as ask reviewers to judge whether the description of materials and procedures are sufficient so that independent research groups can replicate the research. However, when encouraging the research community to replicate and reproduce, editors and reviewers have to reconsider the evaluation criteria for research papers.

Besides the thoroughness of theoretical grounding and methods, novelty and relevance to the field are core evaluation criteria. For instance, *Transactions in Human-Robot-Interaction* asks their reviewers to rate whether the manuscript is interesting and inspiring intellectually and whether its contribution is sufficiently distinct from existing work.

The HRI 2020 conference informed authors that reviewers, amongst other criteria, pay attention to the originality of the work. Other journals, such as the *International Journal of Social Robotics*, have no specific reviewer guidelines. But since novelty, originality, and relevance are promoted pervasively, we can assume that reviewers will have them on the top of their minds when reviewing. Especially repetitions and replications, but also reproductions, are often judged as lacking novelty, originality, and/or relevance (as often defined by the reviewer guidelines). They are, however, highly relevant. A significant contribution is not always a novel study. Replications and reproductions validate and, in the case of the latter, also extend prior work, which is of high importance to science.

Consequently, journal editors and PC chairs have to reconsider the reviewing policies and guidelines to correctly assess the merit of replications. The reproducibility track at HRI 2020, with its own review guidelines, was an attempt to address this issue, but it is not enough. Journal editors and PC chairs should invest into setting up new or redefining existing evaluation criteria. Most importantly, they have to invest into educating and training their reviewers to acknowledge and adhere to these new, more inclusive guidelines.

**Table 2:** Guidelines related to technical challenges

Issue/challenge	Importance	Suggested solutions
**(1)** Physical robot bias (“Physical Robot Bias”)	**(1)** In-person and physical robot studies not always possible (e.g., due to the COVID-19 pandemic).	**(1)** Educate researchers (“Educating and Training Reproducibility Researchers”).
**(2)** Commercial robots being expensive and rigid (“Commercial Robots”).	**(1)** Difficult to modify. **(2)** Costs limit access to existing platforms. **(3)** Reinforces and amplifies biases (“Physical Robot Bias, Classism Bias–Robot Morphology Biases”).	**(1)** Use modular robot units (“Robot Kits”). **(2)** Create custom-designed robots (“Custom-Designed Robots”).
**(3)** Challenges with accessing (multiple/expensive/physical robots for experimentation (“Systems-Related Challenges”).	**(1)** Creates bias in terms of institutions/research groups which creates bias in terms of research participation, recruitment, data, findings and results (“Classism Bias–Robot Morphology Biases”).	**(1)** Secure industry support for reproducibility. **(2)** Encourage simulations/online evaluations or evaluation on previously collected datasets. **(3)** Encourage comprehensive surveys. **(4)** Encourage meta-analyses (“Quantitative Studies”).
**(4)** Lack of shared HRI artifacts (datasets, code, tools) and repositories for collecting and distributing such artifacts (“Software Artifacts”).	**(1)** Impedes (students) quick entry to field. **(2)** Creates barriers of entry for incremental research. **(3)** Does not incentivize “deeper” investigation of concepts/algorithms/data. **(4)** Blocks synergistic impacts of the field. **(5)** Doesn’t encourage accountability.	**(1)** Create guidelines for ethical data collection and sharing. **(2)** Create guidelines for artifact evaluation. **(3)** Encourage usable and modular code. **(4)** Create venues where artifacts are as valued as other research outputs. **(5)** Create HRI repositories for artifacts.

**Table 3:** Guidelines related to HRI research practices

Issue/challenge	Importance	Suggested solutions
**(1)** Researchers overly focusing on novelty/fearing failure/impact on reputation (novelty bias — “Novelty Bias”).	**(1)** Stops nurturing reproducibility efforts/theme. **(2)** See issues listed in Challenge **(1)** in Table 2.	**(1)** Embrace failure (“Embrace “Failure””). **(2)** Encourage novel instruments/concepts for replication/reproduction (e.g., transferring from physical to online). **(3)** Encourage benchmarking events similar to other fields.
**(2)** Unpublished or undisclosed experiments involving systems not working as intended or insignificant results (positive results bias — “Positive Results Bias”)	**(1)** Slows down progress. **(2)** Unfair to students/faculty whose careers depend on publishing. **(3)** Can foster healthy research environment.	**(1)** Embrace failure (“Embrace “Failure””). **(2)** Incentivize papers on negative results. **(3)** Incentivize (short) papers with insightful findings (implemented at HRI 2022). **(4)** Encourage pre-registration of studies with flexibility (“Pre-registration”).
**(3)** Reproducibility for qualitative or design-focused HRI perceived as difficult/challenging/unclear (“Qualitative Studies”).	**(1)** Creates bias in terms of HRI research (themes). **(2)** See issues listed in Challenge **(1)** in Table 2.	For qualitative replication: **(1)** Describe method used, research setting and sampling procedures. **(2)** Document interactions with participants. **(2)** Systematize research results, e.g., with narrative reviews. **(3)** Increase transparency.
**(4)** Sample size bias (“Sample Size Bias”).	**(1)** If sample size bias $< {n}$, research is unworthy.	**(1)** Acknowledge sample size bias and its limitations. **(2)** Educate researchers (“Educating and Training Reproducibility Researchers”).
**(5)** NHT evaluation and test methods (“Null Hypothesis Testing (NHT)/Normative Statistics Bias”)	**(1)** Disadvantages HRI exploratory design work and theoretical algorithmic work that might not have a hypothesis to test.	**(1)** Acknowledge limitations and move away from NHT (“Null Hypothesis Testing (NHT)/Normative Statistics Bias”). **(2)** Educate and train researchers (“Educating and Training Reproducibility Researchers”). **(3)** Rethink guides for authors and reviewers (“Rethink and Restructure Guides for Authors, Reviewers, and Editors”).
**(6)** One-off HRI studies, not grounded in theories of interaction/discouraging replication.	**(1)** Unable to verify results and findings. **(2)** Requires an exercise of reinventing the wheel.	**(1)** Request support materials to enable replication and reproduction. **(2)** Scrutinise support material.
**(7)** Lack of broad interdisciplinarity. Failure to engage experts from fields outside of HRI (e.g., gender studies, critical race theory) in technology and study design.	**(1)** Resulting work may be theoretically unsupported, unethical, or offensive. **(2)** Reinforces and amplifies biases (“Bias in HRI Research Evaluation and Practice”).	**(1)** Increase disciplinary inclusivity in HRI research. **(2)** Reproduce work from outside disciplines with the involvement of the original researchers.

## Summary and Conclusion

This article identifies numerous important issues that the lack of focus on reproducibility creates in the field of HRI research. It also highlights why these issues are critical to the success of our research community and to furthering the science of HRI. Whenever possible, it also attempts to offer a variety of solutions, based both on the experiences of HRI researchers and on best practices that may be adopted from related fields. In order to present these suggestions in a concise manner that allows the reader to quickly grasp the scope of these issues and their interrelationships, Tables 2 and 3 summarize HRI research practices, respectively, as guidelines.

This review serves as an introduction to and overview of reproducibility in HRI, highlighting challenges that exist for supporting reproducible research. The Reproducibility theme at the HRI 2020 conference is discussed as an example of the breadth of HRI research which is already addressing these challenges and foster opportunities to engage with reproducibility in ways that are relevant to all aspects of this highly interdisciplinary field. Challenges to the field of HRI arise from its technical focus and from sources of bias in research practices themselves. Changes to the way HRI researchers are trained could lead to a research community that values and practices reproduction. The authors propose solutions to address these varied challenges, based on both their experiences as HRI researchers and on best practices from related fields.

## Footnotes

1 http://humanrobotinteraction.org/2020/full-papers/

2 http://doc.aldebaran.com/2-5/dev/cpp/

3 http://doc.aldebaran.com/1-14/software/choregraphe/choregraphe_overview.html

4 https://neurips.cc/Conferences/2020/Schedule?showEvent=16158

5 https://www.cos.io/our-services/registered-reports#journals
